# The Relationship Between Blood Monocyte Count and Coronary Artery Ectasia

**DOI:** 10.14740/cr315w

**Published:** 2014-10-06

**Authors:** Mehmet Demir, Canan Demir, Serdar Keceoglu

**Affiliations:** a Bursa Yuksek Ihtisas Education and Research Hospital Cardiology Department, Bursa, Turkey; b Bursa Sevket Yilmaz Education and Research Hospital Infectious Disease Department, Bursa, Turkey

**Keywords:** Coronary artery ectasia, Monocyte, Inflammation, Endotel dysfunction

## Abstract

**Background:**

The pathophysiology of coronary artery ectasia (CAE) has not been clearly identified, although multiple abnormalities including arteritis, endothelial dysfunction, and atherothrombosis have been reported. It is known that monocytes play an important role in inflammation, atherosclerosis and cardiovascular disease. We aimed to compare the numbers of monocyte counts of the CAE patients versus controls.

**Method:**

This study included 84 CAE patients (40 male, mean age 55.4 ± 9.7 years) and 30 controls (10 male, mean age 57.86 ± 11.6 years). Concurrent routine biochemical tests and neutrophil, lymphocyte, monocyte count and mean platelet volume (MPV) on whole blood count were performed for these participants. These parameters were compared between groups.

**Results:**

Baseline characteristics of the study groups were comparable. CAE patients had a higher MPV value and monocyte count than controls (8.8 ± 0.2 vs. 6.2 ± 1.6 fL and 732 ± 88 vs. 321 ± 75 cell/μL; both P < 0.001, respectively).

**Conclusion:**

As a result, our study revealed a relationship between monocyte count and MPV in patients with CAE.

## Introduction

Coronary artery ectasia (CAE) has been characterized as a localized or diffuse non-obstructive lesion of the epicardial coronary arteries with a luminal dilation exceeding 1.5-fold the normal adjacent segment or vessel diameter [1]. The prevalence of CAE varies from 1.2% to 4.7% among patients undergoing coronary angiography [2-5].

The etiopathogenesis of this coronary enlargement is completely unknown. Although the exact mechanisms leading to CAE are not clear up to now, atherothrombosis and endothelial dysfunction, and vasculitis have been suggested as possible responsible factors. CAE has also been reported in association with various conditions such as congenital coronary anomalies, connective tissue diseases, and vasculitis [6, 7].

It is known that monocytes play an important role in inflammation and atherosclerosis [8]. Recent studies have demonstrated increased numbers of circulating monocytes in individuals with prevalent atherosclerotic disease and also monocyte count can independently predict cardiovascular events [9-11].

The powerful inflammatuar and atherosclerotic effects of monocytes made us hypothesize that there might be a correlation between monocyte concentration and CAE. As far as we know, there is no study performed until today about the association of blood monocyte concentration with CAE. In our study, we compared monocyte counts, between CAE patients and control groups.

## Materials and Method

The study group included 84 patients (40 male, mean age 55.4 ± 9.7 years) with isolated CAE who had irregularities with ectatic coronaries without any stenotic lesions under visual assessment. The control group consisted of 30 age- and gender-matched subjects (10 male, mean age 49.16 ± 9.2 years) who proved to have normal coronary angiograms. The indication for coronary angiography was either the presence of typical angina or positive or equivocal results of noninvasive screening tests for myocardial ischemia in both of the groups.

Physical examination, medical history of patients, blood biochemistry and transthoracic echocardiographic examination were evaluated in both groups to exclude systemic diseases. Patients with obstructive coronary artery disease (who had coronary stenotic lesions of > 20%), chronic renal failure, chronic liver disorders, chronic lung disease, moderate or severe valvular disease, hypertension, diabetes mellitus, congenital heart disease, left ventricular systolic dysfunction on echocardiography (EF < 50%), anemia, pregnancy, obstructive sleep apnea, hematological disorders, known malignancy, thyroid dysfunction, hypercholesterolemia, electrolyte imbalance, and drug history including anti-gout agent, antiinflamuar agent (steroid or nonsteroid), antiaggregant or anticoagulant agents, antihistaminic and any medication that can potentially interfere with the measurement of monocyte counts were excluded from the study. Patients who had a recent history of an acute infection or/and high body temperature > 38 °C, and an inflammatory or allergic disease were also excluded from the study.

The patients having a systolic blood pressure ≥ 140 mm Hg and/or a diastolic blood pressure ≥ 90 mm Hg and those taking antihypertensive drugs were accepted to be hypertensive. Diabetes was defined as a fasting blood glucose level > 126 mg/dL or current use of a diet or medication to lower blood glucose. Current cigarette smoking was defined as use of > 10 cigarettes/day at the time of diagnosis.

### Coronary angiography

Coronary angiograms were performed with a femoral approach using the Judkins technique without the use of nitroglycerin, adenosine, or a calcium channel blocker. All patients in the study population underwent elective coronary artery angiography using Siemens Axiom Artis DFC (Siemens Medical Solutions, Erlangen, Germany) following appropriate patient preparation. Coronary angiograms were judged with regard to smooth appearance, luminal wall irregularities, epicardial local or diffuse caliber reduction, and stenosis. CAE was defined as dilation of the coronary artery > 1.5-fold the diameter of the adjacent normal coronary vessels according to Oliveros et al [3].

### Laboratory tests

Biochemical parameters were analyzed spectrophotometrically on ArchitectC16000 (Abbott., USA) autoanalyzer using enzymatic-colorimetric assay.

For whole blood count (monocyte count, hematocrit, hemoglobin, MCV, MPV, leukocytes, neutrophil, lymphocyte and platelets), the blood samples were collected in tubes with EDTA and analyzed on CELL-DYN 3700 (Abbott., USA) device using impedance and optic scatter method.

### Statistical analysis

SPSS 16.0 statistical program (SPSS Inc., Chicago, IL, USA) was used for statistical study. All values are given as mean ± standard deviation. Mean values of continuous variables were compared between groups using the Student’s *t*-test or Mann-Whitney U test, according to whether normally distributed or not, as tested by the Kolmogorov-Smirnov test. A P value of less than 0.05 was considered significant.

## Results

Evaluating basic clinical and demographic characteristics, there was no statistically significant difference between two groups in terms of age, gender distribution, body mass index, and smoking status and biochemical parameters (Table 1).

**Table 1:** Comparison of Basic Clinical and Biochemical Features of Patients and Controls

	Patients (n = 84)	Controls (n = 30)	P value
Age (years)	55.4 ± 9.7	57.8 ± 11.6	NS
Sex (n, %) males	40 (50%)	10 (33%)	NS
Body mass index (BMI) (kg/m^2^)	28.8 ± 5.3	28.5 ± 4.6	NS
Smoking	18 (18%)	6 (20%)	NS
Fasting glucose (mg/dL)	96.3 ± 9.2	97.6 ± 8.5	NS
Creatinin (mg/dL)	0.7 ± 0.1	0.72 ± 0.2	NS
Total cholesterol (mg/dL)	218 ± 46	181 ± 36	NS
Trigliserid (mg/dL)	165 ± 57	151.9 ± 41	NS
TSH (µIU/mL)	1.7 ± 0.6	1.6 ± 0.4	NS

TSH: thyroid-stimulating hormone; NS: nonsignificant.

Given blood count parameters, in the group of CAE patients, blood monocyte count, neutrophil count and MPV value were significantly higher, and lymphocyte counts were significantly lower in comparison with the control group. There was no statistically significant difference between two groups with regard to leukocyte count, platelet count, hemoglobin and hematocrit level (Table 2).

**Table 2:** Comparison of Whole Blood Count Features of Patients and Controls

	Patients (n = 84)	Controls (n = 30)	P value
Hemoglobin (g/dL)	13.7 ± 2.1	13.49 ± 1.15	NS
Hematocrit (%)	41.1 ± 3.5	40.7 ± 3.49	NS
Platelet (10^3^/μL)	231 ± 76	234 ± 60	NS
Leukocyte (/μL)	7,202 ± 455	7,723 ± 546	NS
Monocyte count (/μL)	732 ± 88	321 ± 75	< 0.001
Neutrophil count (/μL)	561 ± 29	356 ± 18	< 0.001
Lymphocyte count (/μL)	282 ± 12	453 ± 32	< 0.001
Mean platelet volume (MPV) (fL)	8.8 ± 0.2	6.2 ± 1.6	< 0.001

Monocyte count was found to be significant parameter using logistic regression analysis (OR: 1.113, 95% CI: 0.564 - 1.662, P < 0.001).

## Discussion

In our study, we have found significant differences in peripheral blood monocyte count and MPV between CAE patients and control group.

The pathophysiology of CAE has not been clearly identified yet, although multiple abnormalities including inflammation, endothelial dysfunction, vasculitis, and atherothrombosis have been reported [5]. CAE is in association with connective tissue disorders such as scleroderma, in Ehlers-Danlos syndrome, also in syphilitic aortitis and in Kawasaki disease [12].

Previous studies have demonstrated that CRP and neutrophil lymphocyte ratio were higher in patients with CAE than in control participants. The increased levels of CRP and neutrophil lymphocyte ratio may suggest that these markers may be used in clinical practice for the assessment of the inflammatory status of CAE [13-15].

As far as we know, there is no study available in the literature about the association between CAE and blood monocyte count.

In our study, we have found significant differences in MPV between CAE patients and control group. Additionally neutrophil counts were higher, and lymphocyte counts were lower in the patients with CAE. Also, our findings are consistent with previous studies [16-18].

Additionally when two groups were compared in our study, monocyte counts of the patients having CAE were significantly higher than control groups.

Monocytes and subtypes have been recognized to possess different migratory rates and functions in inflammatory tissue damage [19]. In monocytes, CD143 (membrane-bound ACE enzyme) expression increases during differentiation toward macrophages and dendritic cells [20].

Previous studies demonstrated the presence of infiltrating monocyte/macrophage cell in the damaged aortic wall of aortic aneurysm patients [21, 22]. Also Ghigliotti et al showed that patients with abdominal aortic aneurysm had higher percentage of circulating monocyte subtypes (CD14^+^ CD16^+^) than controls. These findings suggest that monocytes and circulating subtypes are involved in vascular injury, and also monocytes may affect cardiovascular system through inflammatory cell infiltration [23].

Recent studies showed that the expressions of cellular adhesion molecules including monocytes in patients with CAE are higher than controls [24, 25].

Sahin et al showed the relationship between CAE and neopterin which shows monocyte/macrophage activation [26]. Similarly, Iizuka et al showed the relationship between CAE and urinary neopterin concentration in patients with Kawasaki disease [27].

The powerful inflammatuar, atherogen and vasoactive effects of monocytes made us hypothesize that there might be a correlation between blood monocyte concentration and CAE. In the literature, there is no study investigating the association between CAE and peripheral blood monocytes. Our study is of importance with regard to this matter; we investigated the effect of monocyte concentration on CAE patients.

The most important restriction of our study is the limited number of patients. Further studies are required to determine the relation between monocyte count and CAE.

Our results may contribute to etiopathogenesis of CAE and pathophysiological mechanisms of increased prevalence of cardiovascular morbidity and mortality risk in these patients. Increased concentration of monocyte might be explained with vascular destruction, inflammation, endotelial dysfunction and atherothrombosis in CAE patients.
